# A Novel Approach for Partial Discharge Measurements on GIS Using HFCT Sensors

**DOI:** 10.3390/s18124482

**Published:** 2018-12-18

**Authors:** Armando Rodrigo Mor, Luis Carlos Castro Heredia, Fabio Andres Muñoz

**Affiliations:** Electrical Sustainable Energy, Delft University of Technology, 2600 GA Delft, The Netherlands; A.RodrigoMor@tudelft.nl (A.R.M.); F.A.MunozMunoz-1@tudelft.nl (F.A.M.)

**Keywords:** partial discharges, GIS, SF_6_SF_6_, HFCT, corona discharges, HF, VHF, UHF, antennas, insulation, high voltage

## Abstract

This paper presents a novel measuring system for partial discharge (PD) measurements in Gas Insulated Systems (GIS) using high frequency current transformers (HFCT). The system is based on the measurement of the induced PD currents in the GIS enclosure. In opposition to the existing antenna technologies that measure the radiated energy in the very high frequency/ultra-high frequency (VHF/UHF) range, the proposed system measures the PD conducted currents in the high frequency (HF) range and below. The foundation of the measurements together with a detailed explanation of the sensor installed conveniently at the bolts of the GIS spacer are presented. An experimental study on the current distribution in the GIS enclosure is described to evaluate the impact of the sensor on the measurements. Laboratory experiments have been performed that show the suitability of this method to properly measure particle discharges caused by corona, surface and free moving particle discharges in SF_6_. Discharges in the range of 1 to 4 pC have been properly measured. An analysis to evaluate the performance of the method is shown, in comparison to VHF/UHF antenna measurements. The potential benefits of this novel technique rely on the small attenuation of PD signals in the GIS components in the HF range and sample rate reductions. Finally, a discussion on the potential applicability of present cluster and charge calculation techniques to the proposed PD GIS measurement using HFCT is presented.

## 1. Introduction

Gas insulated systems (GIS) are relevant systems for the delivery of electrical energy, the functionality of which can be endangered by defects in their electrical insulation. Partial discharge measurement has long been an effective tool for monitoring and diagnostics of high voltage (HV) GIS insulation. An extended methodology is to use antennas that pick up the electromagnetic field produced by partial discharges. The vast majority of measurements of this type are performed in the VHF (very high frequency: 30 MHz–300 MHz) and the UHF (ultra-high frequency: 300 MHz–3000 MHz) frequency range.

The GIS enclosure acts as a cavity resonator in which different propagation modes are excited. In turn, these modes can be picked up by antennas installed in the GIS enclosure. The GIS enclosure also acts as a Faraday cage that shields the antennas from external electromagnetic interferences and enables a low background noise level, resulting in a high sensitivity of the UHF method. The UHF method is used extensively because is less sensitive to noise from inverter control systems and power supplies, components that adds disturbances in the Hz to kHz band [1]. In addition, narrow band filtering in the VHF range may allow a better noise suppression improving sensitivity in noisy environments.

On the other hand, in the frequency range of the VHF/UHF is not possible the estimation of the partial discharge (PD) charge nor to follow a charge calibration procedure because the low-frequency components (towards DC) of the PD pulse are not measured by the VHF/UHF sensors. As a result, documents as the IEC TS 62478 [2,3] suggest rather a “sensitivity check” as a means to verify that the complete measuring system on-site is able to pick up signals equivalent to 5 pC in a section between two adjacent sensors as measured by the conventional IEC 60270 method during laboratory tests. Recent outputs from the CIGRE JWG D1/B3.57 still attach to this practice, recommending PD testing with AC voltages.

However, with the growing trend towards HV DC GIS technology, testing with DC voltages, development of evaluating tools such as the normalized and differenced values from ∆Δt and PD magnitudes (NoDi) patterns as an alternative for PRPD patterns [4] and new measuring techniques are gaining renewed relevance. In the field of measuring techniques, the optical detection is a technique that has caught attention due to its resilience to noise and ability to detect glow-less PD signals that usually cannot be detected by electrical methods.

In this paper, an alternative measuring system is introduced, which makes use of HFCT sensors. The proposed methodology measures the current induced in the enclosure by the PD phenomena. The HFCTs are installed at the bolts of the spacers, in such a way that they are able to measure the current travelling along the GIS compartments. Unlike the antennas, the HFCT does not measure the electromagnetic field in the insulation, but the induced currents in the enclosure by a PD event.

In the GIS, the electromagnetic waves produced by a PD pulse propagate as in a coaxial transmission line. Each GIS component such as spacers, T-shape branches, etc., has his own electromagnetic behavior. As a result, the electromagnetic waves suffer from damping and dispersion as they travel along the different components, being the damping and dispersion particularly high in the VHF and UHF range. Accordingly, the bandwidth of the HFCT presented in this paper is chosen from hundreds of kHz to a few hundred MHz, where the attenuation due to the GIS components is relatively smaller in comparison to the VHF and UHF range [5]. Taking advantage of this lower attenuation, the measuring system here introduced aims to offer an increased spatial sensitivity as compared to VHF/UHF systems.

In the following sections, this paper will provide a feasibility study and performance of the HFCT-based measuring system for GIS. Section 2 describes the actual-size GIS that was used as test object and the instrumentation. Section 3 and Section 4 are intended for the description of the sensor installation and arrangement to measure the currents flowing along the GIS compartments. Section 5 presents the distribution of the PD currents in the GIS compartments. Next, in Section 6, a sensitivity check procedure is reported and compared to measurement results from a VHF/UHF system. Finally, discussion about the HFCT system and conclusion is presented in Section 7 and Section 8 respectively.

## 2. Test Object and Set-Up

**Figure 1:**
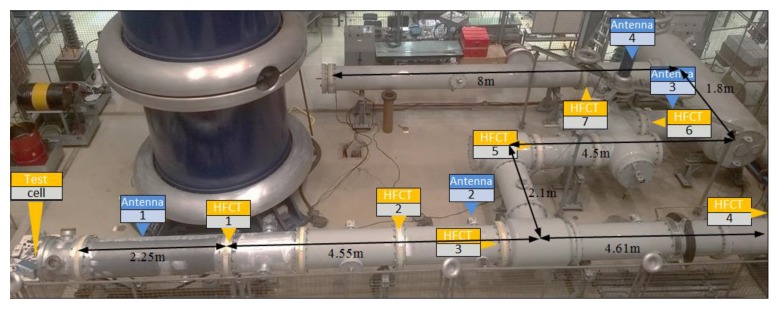
Test object indicating the location of ultra-high frequency (UHF) and high frequency current transformers (HFCT) sensors.

The experiments reported in this paper were conducted on a 380 kV, SF_6_, actual-size, GIS available at the High Voltage Laboratory of TU Delft, see Figure 1. This GIS spans over an area of approximately 11 × 6 m and it includes spacers, T-joints, earth switch, switchgear, bushing and a disconnector. The labels correspond to seven of the spacers where HFCT sensors were installed. The location of the UHF antennas is also indicated.

Two types of measurements were conducted on the GIS: measurements with injected signals and PD measurements with a set of three test cells under SF_6_ pressure to produce corona, surface and free moving particle discharges.

**Figure 2:**
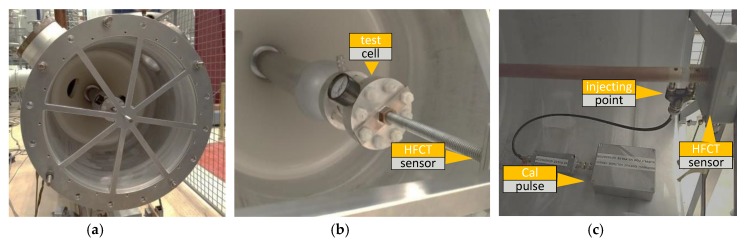
Gas insulated system (GIS) end, (**a**) ground electrode; (**b**) positioning of the test cell and sensor; (**c**) positioning of pulse calibrator.

For the measurements with injected signals, the GIS end was given a special preparation in which a rod with a proper connector is at one side threaded in the grounded GIS lid (Figure 2a) and at the other side connected to the GIS main conductor (Figure 2c). For PD measurements, this rod is removed and a test cell is put in its place. With this configuration, the ground electrode of the test cell is connected to the GIS enclosure via a lid, see Figure 2a, having multiple current return paths. An HFCT, Figure 2b, is installed at the rod holding the test cell to measure the PD current injected in the GIS.

## 3. The HFCT-Based Measuring System for Gas Insulated System

When a PD event occurs, at sufficiently high frequencies, the transverse electric (TE) and transverse magnetic (TM) are the predominant propagation modes of PD electromagnetic waves and each of these modes has a cut-off frequency below which it will not propagate [5,6,7].

The propagation in higher order modes is dispersive in nature, resulting in that a single input pulse is transformed into a damped oscillatory signal [5]. For instance, in [8] is reported that the damping of the PD pulse is frequency-dependent, with frequency components above 500 MHz remarkably damped by spacers, T-shape branches and E-bends. In addition, the damping in the PD signals is due to the finite conductivity of the conductors and the dielectric losses [7].

**Figure 3:**
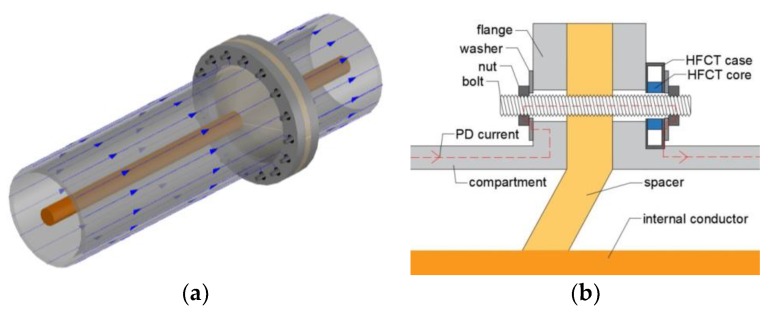
(**a**) Partial discharge (PD) pulse currents as they travel along the GIS; (**b**) PD pulse current flowing along the bolts connecting two compartments.

At lower frequencies in a GIS, the preponderant mode of propagation is the transverse electromagnetic (TEM) mode. In the range of dozens of kHz to hundreds of MHz, the PD pulse creates a fast surface current which travels along the inner part of the compartments and the outer part of the main conductor. This PD current flows mainly on the surface of the conductors due to the skin effect, and it suffers less distortion and attenuation than at higher frequencies. This behavior serves as the hypothesis that: first, a PD event induces currents at the GIS compartments that can be measured by HFCT sensors as illustrated in Figure 3, and second, the lower attenuation of the PD current will lead to picking up signals further away from the PD source, increasing the spatial sensitivity.

In Figure 3a, a spacer is placed in between two compartments. The PD pulse current flows along the compartments and bridges the spacer via the bolts as depicted in Figure 3b, which connect adjacent compartments [9]. Figure 3b shows that the bolts are not in electrical contact with the flange of the compartments, therefore the current flows along the bolts. An HFCT properly installed at one of the bolts of the spacer picks up the magnetic field produced by the PD currents. The HFCT measures a portion of the PD currents since they split over the total amount of bolts. The washers and nuts act like bridges closing the path between the bolts and the compartments.

It is worth mentioning that the spacer-bolt disposition presented in this paper is characteristic of the first GIS designs. Present spacer designs are “internal” spacers where the installation of the HFCT, as described in this work, is not feasible without design modifications.

## 4. HFCT Sensor

An HFCT sensor was chosen as the most suitable option to measure the currents through the bolts given its gain, bandwidth and mechanical properties. Since the PD current magnitude is in the mA range, a high gain is demanded from the sensor. In addition, the lower cut-off frequency has to be the lowest possible, while the upper frequency must be in the range of a few hundred MHz. Meeting these gain and bandwidth requirements the sensor better approximates the PD pulse shape, in turn reducing the errors in PD parameter computation.

**Figure 4:**
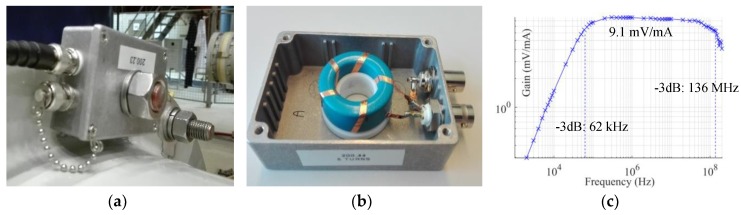
(**a**) Installation of the HFCT; (**b**) construction of the HFCT sensor; (**c**) sensor frequency response.

A picture of the HFCT sensor installed at the bolts of the spacer is observed in Figure 4a with a picture of its teardown in Figure 4b. Its measured frequency response is shown in Figure 4c.

Bandwidth and gain are tied parameters in HFCT designs where a small number of turns increases the gain but decreases the bandwidth [10]. For this application, the requirements of the bandwidth were priority over the gain. Amplifiers were added to the sensors output to step up the sensitivity of the system. The secondary winding of the HFCT sensor comprised five turns wound onto a N30 ferrite core [11]. The five turns were stripes of 3 mm copper tape wound evenly distributed onto the core. The flatness of copper tape and the distance between turns helped reduce the stray capacitances enhancing the response at higher frequencies. The frequency response of the sensors can be observed in Figure 4c. The built HFCT has a gain of 9.1 mV/mA and a bandwidth from 62 kHz to 136 MHz. The sensor has been equipped with an extra BNC connector, that when short-circuited connects the secondary of the HFCT to the GIS enclosure.

## 5. Partial Discharge Pulse Current Distribution in Gas Insulated System

Ideally, the PD current distribution in the GIS spacer should be fully symmetrical, since all the current paths through the spacer rods offer the same impedance. However, when a HFCT is fixed to one spacer bolt, the impedance of the current path is modified due to the input impedance of the HFCT, creating a distortion with respect to the original symmetrical current distribution. Several laboratory measurements were conducted to check the feasibility of the proposed measuring system, and to determine the current distribution in the bolts of the spacer.

The first experimental case corresponded to a measurement where a fast pulse from a calibrator was injected at the GIS end and picked up by HFCT 1 located 2.25 m away in the spacer 1, see Figure 1. In this case, only one bolt was provided with a sensor and the remaining 15 bolts acted as additional current paths, aka configuration 1.

**Figure 5:**
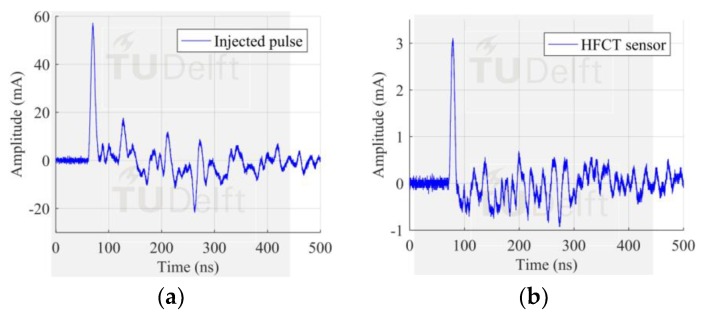
Results with configuration 1, (**a**) pulse injected at the test cell position; (**b**) pulse measured by HFCT 1. Only one sensor installed and 15 parallel current paths.

Comparing both the injected and the measured signal in Figure 5, configuration 1 yielded to pick up 5.43% of the peak value of the injected signal. The theoretical value accounts for 6.25% of the injected current, given that in our case the current is split into 16 paths. This small deviation is due to the extra impedance added by the HFCT to the current path.

Configuration 2 consisted of 4 sensors installed at the spacer 1. In this configuration, 4 bolts symmetrically distributed were equipped with sensors whereas the remaining 12 bolts were acting as parallel current paths. There were 3 bolts in between two sensors which kept the arrangement symmetric.

**Figure 6:**
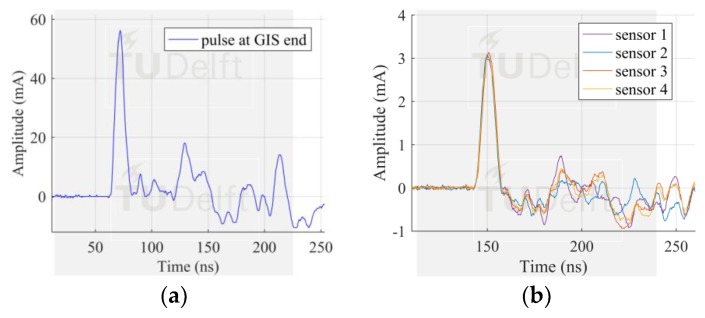
Results with configuration 2, (**a**) pulse injected at the test cell position; (**b**) pulses measured by HFCT 1. Four sensors installed and 12 current paths (remaining bolts).

The similarity in pulse shape and peak amplitude of the measured pulses, showed in Figure 6b, proved that the pulse current flows homogenously along the GIS compartment perimeter. Moreover, the ratio of the peak amplitudes was 5.51%, similar to that achieved by configuration 1.

**Figure 7:**
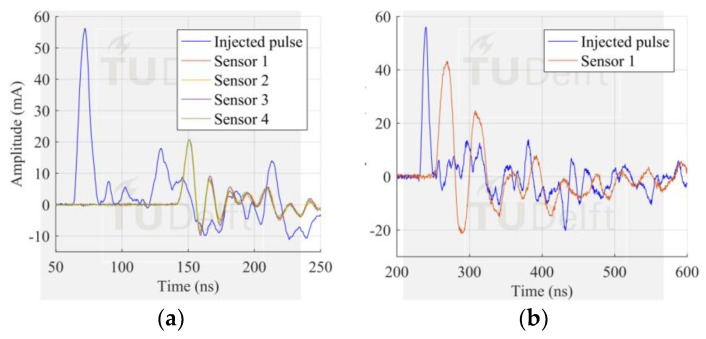
Results with configuration 3, pulse injected at the test cell position (**a**) four sensors installed and no current flowing through the remaining 12 bolts; (**b**) one sensor installed and no current flowing through the remaining 15 bolts.

In configuration 3, the 12 bolts from configuration 2 were given dielectric washer, so that the pulse current now is pushed to flow only along the 4 bolts having sensors. Results from this configuration are shown in Figure 7.

Several differences were found in the results as compared to the two previous configurations. First, the current amplitude measured at each sensor was 37% of the amplitude of the injected pulse at the GIS end. This ratio is significantly higher than the limit assuming that the pulse current is to be split into the 4 sensors which would ideally result in a ratio of 25%. Second, the pulse shape measured by the sensors featured an undershoot not seen in the previous configurations. Unlike configuration 1 and 2, where the amount of parallel conducting bolts was predominantly bigger than the amount of bolts with sensors, in configuration 3 the coaxial transmission line structure of the GIS is broken because the current is forced to flow only through very few or just one path. While in configuration 1 and 2 the effect of the sensors may be negligible causing that the measured amplitudes fit reasonably the simple model of the input current being split into the number of current paths, in configuration 3 adding more sensors and reducing the remaining current paths leads to a big impedance change and in turn reflections that distorts the shape of the measured pulses.

Additional evidence is presented in Figure 7b. In this case, just one sensor was left and the remaining 15 bolts were given dielectric washers. The distortion of the measured pulse is such that its peak amplitude is around 70% of the injected pulse amplitude. Furthermore, it shows a significant distortion, with undershoot and pulse width increase.

According to the results of the experiments, configuration 1 is the preferred measurement arrangement because the effect of reflections is minimum as compared to other configurations. This is a critical factor if estimation of PD quantities is the target of the study. Thus, this configuration is the one used in the following PD tests and sections. Configuration 2 does not offer an advantage over configuration 1 since it was proven that the distribution of the PD current is homogenous in the perimeter of the compartment. Configuration 3 is strongly affected by reflections although improves sensitivity.

## 6. Performance Analysis of the HFCT-Based Measuring System for Gas Insulated System

The performance of the HFCT-based measuring system to detect typical PD defects was assessed by means of a special setup arrangement. The setup used four different sensors as described:

- Sensor 1. The first sensor was located at test cell position to measure the total PD current injected in the GIS. A fast current transformer FCT-016-5.0 from Bergoz was used for this purpose. This sensor has a bandwidth of 3.92 kHz − 1.11 GHz that in combination with a sampling rate of 6.25 GS/s allowed for accurate measurements of the PD charge at its source. Charge estimation was performed according to [12,13] and used as a reference. Please note that the charge is not estimated according to IEC60270. The trigger of the measuring system was done using this signal.

- Sensor 2. A HFCT transformer as described in Section 4 was installed at position HFCT 1, at 2.25 m from the source, see Figure 1. Peak values of the recorded signals were evaluated.

- Sensor 3. Another HFCT, equal to sensor 2, was installed at position HFCT 7. The location of this sensor is far away from the test cell position, at a distance of 16 m. Peak value of the recorded signals were evaluated.

- Sensor 4. A VHF/UHF antenna installed at location Antenna 1, see Figure 1. The antenna was used as reference to the present PD detection methods in GIS.

To check the performance of the HFCT measuring system, each test cell was tested and all sensor signals recorded. The sensor at the source was used as the trigger source and for charge evaluation purposes. All signals were recorded simultaneously. Details of the circuit developed to acquire the phase of the PD pulses can be found in [11]. The analysis of the signals from the HFCTs and the comparison with the antenna are reported in the following chapters. Before presenting the performance analysis, the PRPD patterns for each defect are shown next.

### 6.1. PRPD Patterns of Test Cells

**Figure 8:**
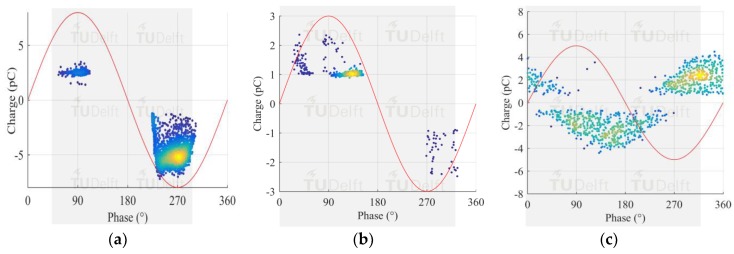
(**a**) Corona discharge; (**b**) Surface discharge; (**c**) Free moving particle.

Results, reported in Figure 8, show that representative PRPD patterns were recorded for each different type of defect.

The test voltage and SF_6_ pressure were adjusted for each test cell to produce small discharges, in the range of a few pC, suitable to check the performance of the proposed measuring system. Small magnitudes in the order of 1 to 4 pC were attained.

### 6.2. Sensitivity Check

The sensitivity attainable by the measuring system in configuration 1 was determined by its ability to pick up the signals corresponding to the PD tests reported in the previous section (Figure 8). The smallest and the largest signal from each PD test were chosen as case of study. The measured signal by the antenna 1 and HFCT 1 were compared for the case of the smallest signals from each PD tests. The largest signals were used to verify the sensitivity of the HFCT sensors at different locations. In addition, to contrast the performance of the system, the results were compared to the results of a VHF/UHF measurement. Details of the VHF/UHF sensors can be found in [14]. Different amplification ratios were necessary in each test. Table 1 reports the testing parameters and specifications of amplifiers and sensors.

**Figure 9:**
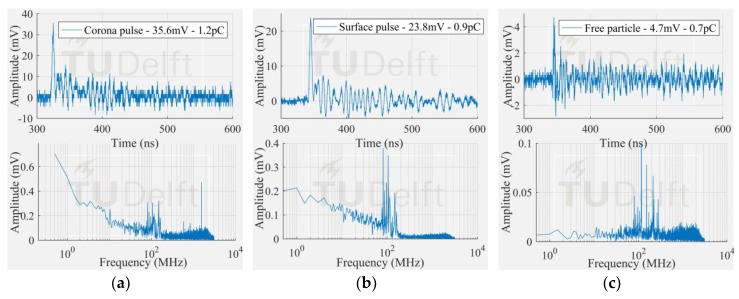
Smallest pulse at the test cell (**a**) corona; (**b**) surface; (**c**) free moving particle.

Figure 9 shows the smallest signal and its corresponding frequency spectrum from each PD test. An indication of the magnitude in pC after integration of the current signal [12] is also given.

**Figure 10:**
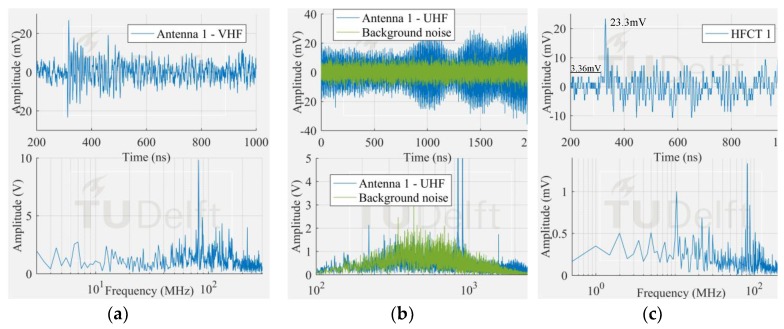
Smallest corona signal measured by (**a**) antenna 1 in VHF; (**b**) antenna 1 in UHF; (**c**) HFCT 1.

**Figure 11:**
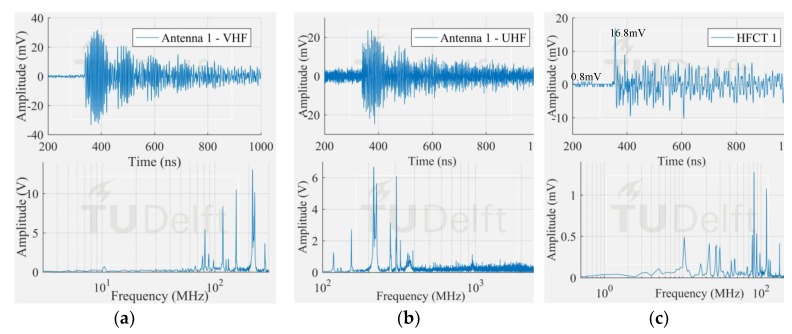
Smallest free moving particle signal measured by (**a**) antenna 1 in VHF; (**b**) antenna 1 in UHF; (**c**) HFCT 1.

**Figure 12:**
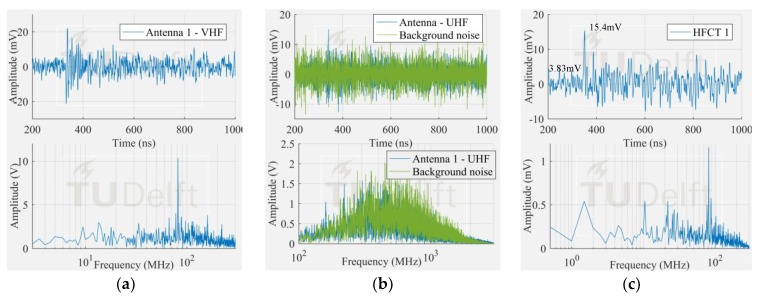
Smallest surface signal measured by (**a**) antenna 1 in VHF; (**b**) antenna 1 in UHF; (**c**) HFCT 1.

The voltage signals corresponding to the smallest PD pulses in Figure 9 measured by the antenna 1 and HFCT 1 are observed in Figure 10, Figure 11 and Figure 12.

The corona signal can be distinguished in the VHF range and by the HFCT 1 but not in the UHF range. In the UHF range, the signal from the antenna is affected by the noise picks appearing at around 850 MHz.

The surface test case was similar to the corona test case. The measured frequency content of the surface discharge is significant up to around 100 MHz, and as a result, the antenna 1 in VHF picked up the discharge signal but not in UHF range. On the other hand, the free particle discharge produced a frequency spectrum that extended mainly up to 400 MHz, also with a frequency peak at 950 MHz. This broader frequency spectrum resulted in the discharge signal being picked up both in VHF and UHF range.

It is also interesting to notice that the HFCT 1 was able to pick up the discharge signal in all the three study cases. Moreover, the ratio of the peak amplitude and the background noise (peak amplitude before the starting of the pulse) was always higher than 4. This result is remarkable because the vertical range of the 8-bit oscilloscope used for the experiments was set for proper acquisition of the biggest signals, hence unavoidably coursing the digitalization of the smallest ones.

**Figure 13:**
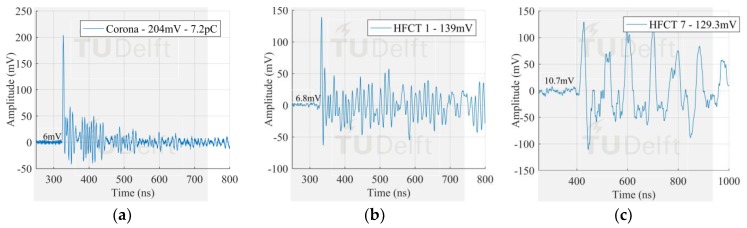
Largest corona signal measured by (**a**) Test cell HFCT; (**b**) HFCT 1; (**c**) HFCT 7.

**Figure 14:**
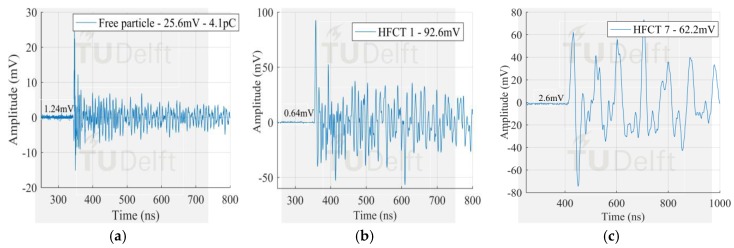
Largest free moving particle signal measured by (**a**) Test cell HFCT; (**b**) HFCT 1; (**c**) HFCT 7.

**Figure 15:**
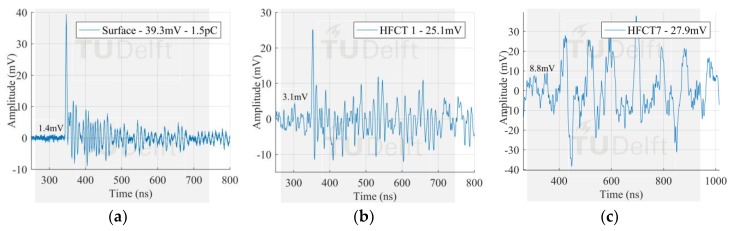
Largest surface signal measured by (**a**) Test cell HFCT; (**b**) HFCT 1; (**c**) HFCT 7.

The signals measured by the HFCT sensors corresponding to the largest PD discharges in Figure 8 can be seen in Figure 13, Figure 14 and Figure 15. In this case study, the signal measured by HFCT 7, located at the opposite end of the GIS (16 m away from the test cell) is also reported. Before reaching the HFCT 7, the induced PD currents passed over the several components of the GIS that distorted the signals. However, the amplitude is reduced in a much less extend as evidenced by the low background noise. On the other hand, fewer GIS components between the PD source and the HFCT sensor results in a lower pulse distortion as can be observed by the results of HFCT 1.

## 7. Discussion

The methodology presented in this paper has lead to successful measurements in laboratory conditions. In a next stage, the performance of this system has to be checked in field conditions were noise floor levels can affect the measurements.

It is worth mentioning that the HFCTs can only be installed in GIS designs featuring external spacers like the one used in this paper. GIS designs, in which the spacer is embedded into the enclosure, will need a mechanical modification to properly allocate the HFCT. For instance, usually a part of a GIS of new design is a compensator. The compartments at both sides of the compensator are electrically connected by copper bars and bolts, resembling the current paths in the type of spacers used in this paper. Thus, the HFCT sensors can be installed at the compensator bars in order to apply the measuring system here introduced.

On the other hand, since the PD pulses are measured with a lower cutoff frequency in the kHz range, charge calculation can be potentially possible. The influence of the partial reflections at the GIS components and a detailed study on the current distribution under the influence of the HFCT transferred impedance has to be properly addressed.

Moreover, PD measurements in the HFCT range suffer from relative small attenuation in comparison with VHF/UHF measurements, which could develop into more sensitive measurements.

It is worth mentioning that GIS grounding does not affect the HFCT-based measuring system, since PDs propagating in the GIS always follow the coaxial structure.

Lastly, traditional [15,16,17] and new post-processing techniques [18,19] could be used to potentially distinguish between real PD sources and external induced noise. Since these techniques have been developed for application in the HF measurement range, extrapolation to GIS application should be possible.

## 8. Conclusions

In this paper, a new partial discharge measuring system for GIS based on HFCT sensors is introduced. The novelty behind this system is that it makes use of HFCT sensors to pick up the PD current induced at the enclosure of the GIS compartments. Through measurement results, it is proven that upon a PD event an induced current pulse travels along the GIS compartments and the current distribution is homogenous in the compartment perimeter. As a result, HFCT sensors properly installed at the bolts of the spacers can measure PD signals with enough sensitivity. It has been demonstrated that when the HFCT is designed with a low lower cut-off frequency and wide band, the sensitivity of the system is high enough as to pick up signals far away from the PD source, leading to a high coverage of the GIS per sensor installed.

In addition, the effect of the different components of the GIS, such as the switchgear, T-joints, spacers, etc. decreases as the sensor is located closer to the PD source. As a validation, the experimental results showed that the signal measured by the HFCT sensor is correlated with the signal measured at the PD source. This is an outstanding result because it opens the possibility to approximate the PD magnitude in pC, which is not possible with VHF/UHF systems.

Other advantage obtained by this system is that a full implementation may be less expensive compared to VHF/UHF systems because the HFCT bandwidth in the range of MHz allows for lower sampling rates which can reduce the cost of the monitoring system.

## Figures and Tables

**Table 1:** Testing parameters used in each test cell.

	Corona	Surface	Free Moving Particle
HFCT Test cell	Sensor: 5 mV/mA, BW 3.92 kHz −1.11 GHz Amp: 26dB, BW 100 kHz −1.3 GHz		Sensor: 9.1 mV/mA, BW 62 kHz −136 MHz
HFCT 1	Sensor: 9.1 mV/mA, BW 62 kHz −136 MHz Amps: 21.7dB, 27 kHz-955 MHz + 25.1dB, 24 kHz −1.14 GHz		Sensor: 9.1 mV/mA, BW 62 kHz−136 MHz Amp: 25.1dB, 24 kHz −1.14 GHz
HFCT 7	Sensor: 9.1mV/mA, BW 62 kHz −136 MHz Amps: 22.7dB, 30 kHz −1.23 GHz + 25.3Bb, 23 kHz −1.23 GHz		Sensor: 9.1 mV/mA, BW 62 kHz −136 MHz Amp: 22.7dB, 30 kHz −1.23 GHz
Antenna 1	Sensor: VHF/UHF Amps: 25.1dB, 21 kHz −1.21 GHz + 25.3dB, 23 kHz −1.23 GHz		Sensor: VHF/UHF Amp: 25.1dB, 24 kHz −1.14 GHz
AC Test voltage	15 kV_RMS_	15 kV_RMS_	12 kV_RMS_
SF_6_	3 Bar	3 Bar	2 Bar
